# The Interaction of Health Behaviors and Cardiovascular Diseases: Investigating Morbidity Risks of Disparities in U.S. Adults

**DOI:** 10.3390/healthcare13233072

**Published:** 2025-11-26

**Authors:** Gulzar H. Shah, Suhail Chanar, Stuart H. Tedders, Kabita Joshi, Kristina Harbaugh

**Affiliations:** Jiann-Ping Hsu College of Public Health, Georgia Southern University, Statesboro, GA 30460, USA; sc34327@georgiasouthern.edu (S.C.); stedders@georgiasouthern.edu (S.H.T.); kj00742@georgiasouthern.edu (K.J.); kharbaugh@georgiasouthern.edu (K.H.)

**Keywords:** cardiovascular diseases, risk factors, health disparities, heart attack, coronary heart disease, stroke, health behavior

## Abstract

**Background:** Chronic diseases are a significant and escalating public health concern in the United States (U.S.) and globally. Chronic co-morbidities such as heart disease, stroke, diabetes, other cardiovascular diseases, and asthma are major risk factors for death and disability. Behavioral factors such as smoking, alcohol use, sedentary lifestyle, and poor dietary habits are among the major risk factors leading to these chronic diseases. **Purpose:** This study aims to investigate how combinations of unhealthy behaviors are associated with the risk of cardiovascular diseases in various populations. **Methods:** Using data from the 2023 Behavioral Risk Factor Surveillance System (BRFSS), we computed multivariable logistic regression models to assess the association of unhealthy behaviors with the risk of chronic diseases. **Results:** Our results show that compounded score of risky health behaviors such as smoking, alcohol use, and physical inactivity, as well as other covariates such as older age, being male, previously married, living in a rented house, unemployed, living in non-metropolitan counties, having high blood pressure, and high cholesterol, were associated with experiencing a heart attack, coronary heart disease, and stroke. **Conclusions:** Our results highlight the need for behavior-focused population health interventions to lower morbidity and health inequities by showing that unhealthy behaviors and sociodemographic disparities significantly raise the risk of cardiovascular diseases.

## 1. Introduction

Non-communicable diseases (NCDs) are a leading global health concern, responsible for an estimated 41 million deaths each year. Among these, cardiovascular diseases (CVDs) account for the most significant proportion, followed by cancer, chronic respiratory diseases, and diabetes. In 2021 alone, an estimated 19 million people died due to CVDs, with the majority of the deaths occurring in low- or middle-income countries [1].

The Global Burden of Disease (GBD) Study 2021 defines Disability-adjusted life years (DALYs) as “Years of healthy life lost to premature death and disability. DALYs are the sum of years of life lost (YLLs) and years lived with disability (YLDs).” The study calculated DALYs by summing years of life lost (YLLs) and years of life lived with disability (YLDs) for each geography, age group, sex, and year. One DALY represents one lost year of healthy life. It is a frequently used indicator to capture the years of life lost due to premature mortality and years lived with disability due to non-fatal health problems.

Chronic diseases such as CVDs, respiratory diseases including chronic obstructive pulmonary disease (COPD), diabetes, and low back pain caused 617.9 million DALYs in 2021. Of these, CVDs—including ischemic heart disease and stroke—accounted for 348.7 million DALYs (56%) [2]. In the United States (U.S.), CVDs remain the leading causes of death and disability. In 2022, they were responsible for 2.37 million deaths, with 868,273 (36.6%) explicitly attributed to heart diseases and stroke. Other major causes included cancer (608,371; 25.6%), accidents and unintentional injuries (227,039; 9.6%), respiratory diseases (147,382; 6.2%), and diabetes (101,209; 4.3%) [3,4].

The burden of chronic disease is particularly high in the United States (U.S.), where 6 out of 10 adults have at least one chronic condition, and 4 out of 10 have two or more [5]. Between 2017 and 2020, an estimated 127.9 million Americans reported having CVD, resulting in $422.3 billion in healthcare costs [6]. When compared to other conditions, heart diseases present significantly higher morbidity than diabetes (~38.4 million) [7], unintentional injuries (~25 million) [8], chronic obstructive pulmonary disease (COPD) (~16 million) [9], Alzheimer’s disease (~7.2 million) [10], and cancer (~1.9 million) [11].

Evidence suggests that many chronic diseases, including CVDs, can be prevented or mitigated by addressing modifiable risk factors such as tobacco use, excessive alcohol consumption, physical inactivity, and poor nutrition [12]. While age and genetic predisposition are non-modifiable, growing research emphasizes the role of social determinants of health—such as living conditions, income level, employment status, and recreational activities. According to the World Health Organization (WHO), improving access to healthcare, nutritious food, physical activity, and stress management resources may significantly reduce the burden of CVDs [13].

Previous research has established strong links between individual risk behaviors and CVD outcomes. For example, around 47 million Americans use tobacco, and smoking-related diseases claim approximately 480,000 lives annually in the U.S. [14,15]. Smoking contributes to coronary heart disease by damaging arterial walls and reducing blood flow to the heart [16]. Similarly, an estimated 28.9 million Americans are affected by alcohol use disorder (AUD), with 178,000 annual deaths attributed to alcohol-related conditions. Excessive alcohol consumption is also associated with several chronic conditions, including ischemic heart disease and ischemic stroke [17,18,19].

Between 2017 and 2020, 25.3% of U.S. adults were physically inactive. Inactivity was even more prevalent among those with chronic diseases, at 47.6% in individuals with CVD [20,21]. Poor nutrition is another critical factor, contributing to over half a million deaths annually, with CVDs being the most prevalent diet-related condition [22,23]. There is growing recognition in the literature that multiple health risk behaviors often interact to produce compounded adverse health outcomes [24,25]. In the U.S., 95.2% of adults report having one or more risk factors for CVD [26]. CVD is frequently accompanied by co-morbidities such as diabetes and arthritis, with risk behaviors like smoking and binge drinking commonly reported [27].

Although hypertension is a primary risk factor for CVDs, other factors such as family history, diabetes mellitus, food preferences, and smoking also show strong associations with this condition [28,29,30]. Additional studies have found that essential hypertension is significantly linked to modifiable risk factors like insomnia, alcohol dependence, and elevated body mass index [31,32,33]. The prevalence of multiple chronic conditions varies across the U.S., ranging from 37.9% in the District of Columbia to 64.4% in West Virginia. Certain geographic regions, such as the South Atlantic, East South Central, East North Central, West South Central, and Mountain areas, have been identified as at particularly high risk for CVDs and associated co-morbidities [33,34,35].

A recent Centers for Disease Control and Prevention (CDC) study found that 26.5% of U.S. zip code areas bear the highest burden of CVDs [36]. Mortality rates due to CVDs tend to be higher in nonmetropolitan rural counties [37]. Racial and ethnic disparities in CVD prevalence also persist, with a widening gap between the Black and White populations [38,39,40]. Lower-income and less educated populations bear a disproportionate burden of CVD, and significant gender disparities exist as well. In 47 of 53 U.S. states and territories, CVD risk factors were more prevalent among women, and increasing age was consistently linked to higher rates of co-morbid conditions [33,36].

While the individual effects of smoking, alcohol consumption, and physical inactivity on CVD are well-documented, there remains a significant gap in understanding how these behaviors interact to compound health risks, particularly within vulnerable demographic subgroups. Most studies have examined these behaviors in isolation, limiting our understanding of their combined impact. Therefore, this study aims to address that gap by examining how combinations of unhealthy behaviors are associated with CVD risk across diverse populations. The goal is to produce research evidence that can inform public health interventions that target high-risk groups in the U.S.

## 2. Materials and Methods

This research is based on a retrospective cross-sectional study design, using data from the 2023 Behavioral Risk Factor Surveillance System (BRFSS). The BRFSS is conducted by the CDC, using a large, representative sample of adults in the U.S. The BRFSS employs a cross-sectional survey design to collect data on health-related risk behaviors, chronic health conditions, and use of preventive services. The sampling methodology ensures representativeness across states and is stratified by factors such as age, gender, and race/ethnicity. A total sample size of 433,320 respondents is used in this analysis.

### 2.1. Measures

Three dependent variables for this study include reported “heart attack,” “angina or coronary heart disease”, and “stroke”. The variable heart disease in the U.S. adult population was operationalized using the question, (1) (Ever told) you had a heart attack, also called a myocardial infarction? (2) (Ever told) (you had) angina or coronary heart disease? (3) (Ever told) (you had) a stroke? The responses to these questions were measured using a binary response: “Yes” or “No”. The missing or not-reported values were omitted.

Our primary independent variable, risky health behaviors, was measured through a composite score that combined smoking, heavy alcohol use, and lack of exercise (0 = none; 1 = any one of the three; 2 = two or more). The last category, “two or more,” was created because the frequency of the last one, “all three present,” was low, leading to small cell sizes and vague results when 0, 1, 2, and 3 were included without combining 3 with 2. Smoking status originally had four levels: Current Smoker—Now Smokes Every Day, Someday smoker, Former smoker, and Non-smoker. The Heavy Alcohol Variable (No, Yes, Don’t Know/Refused/Missing). Other covariates included: Overweight or Obese calculated variable (No, Yes, Don’t Know/Refused/Missing), High Blood Pressure Calculated Variable (No, Yes, Don’t Know/Refused/Missing), High Cholesterol Calculated Variable (No, Yes, Don’t Know/Refused/Missing), Age Group in Years (18 to 24, 25 to 34, 35 to 44, 45 to 54, 55 to 64, 65 or older), Sex of Respondent (Male, Female), Marital Status (Married or Cohabitating, Previously Married, Never married, Refused/Don’t Know/Not Sure), Education Level (Less than High School, High School Graduate, Some College, College Graduate or Higher, Refused/Don’t Know/Not sure), Own or Rent Home (Own, Rent, Refused/Don’t Know/Not Sure), Employment Status (Employed, Unemployed, Not in the Workforce, Unable to Work, Refused/Don’t Know/Not Sure), Income Level (Less than $25,000, $25,000 to $49,999, $50,000 to $99,999, $100,000 or More, Refused/Don’t Know/Not Sure), Five Level Race/Ethnicity Category (White Only Non-Hispanic, Black Only Non-Hispanic, Other Race Non-Hispanic, Multiracial Non-Hispanic, Hispanic, Don’t Know/Not Sure/Refused), and Health Insurance status (Have Some Form of Insurance, Do Not Have Some Form of Health Insurance, Don’t Know, Refused or Missing Insurance Response).

### 2.2. Analytical Methods

Descriptive statistics such as percentages and confidence intervals were computed to assess the survey participants’ disease status, demographic variables, and modifiable behaviors. We conducted multivariable logistic regression with binary outcomes (coded Yes or No—the reference category) for three dependent variables: Heart Attack, Angina or Coronary Heart Disease (CHD), and Stroke. Sampling weights provided in the BRFSS data were applied to ensure that the results are representative of the U.S. adult population.

Behavioral scores: We combined the variables lack of physical activity (exercise), smoking, and heavy alcohol as behavioral scores 0, 1, and 2. The behavioral score is 0 if none of the behavioral risk factors, smoking, heavy drinking, and physical inactivity, are present. The behavior score is 1 if there is a presence of one risk behavior (smoking or heavy drinking or no exercise) in the participant. The behavior score is 2 if there is a presence of two or more than two risk behaviors (smoking or/and heavy drinking or/and no exercise) in the participant.

## 3. Results

Outlined in Table 1 are the descriptive statistics for all other variables included in the study. There were 433,323 subjects in this study. Among U.S. adults, heart attack was present in 4.19% of the study participants, angina or coronary heart disease was present in 4.07% of the study participants, and stroke was present in 3.39% of the study participants.

Behavioral scores:

Our results suggest that around 30% (127,590) of the study participants had at least 1 risk behavior, which was either smoking, heavy drinking, or lack of exercise, and 5.61% (24,028) had 2 or more than 2 risk behaviors in their lifestyle.

Table 2 presents the multivariable logistic regression analysis results of participants who experienced a heart attack, coronary heart disease (CHD), or angina and stroke.

Table 2 presents the multivariable logistic regression analysis results of participants who experienced a heart attack, coronary heart disease (CHD), or angina and stroke. The primary independent variable, “risky health behaviors was significantly associated with all three dependent variables: “heart attack,” “angina or coronary heart disease,” and “stroke.” Compared to participants with no behavioral risk factors (score = 0), those with a behavioral risk score of 2 (indicating 2 or 3 risk behaviors) had significantly higher odds of reporting a heart attack (AOR = 1.53, 95% CI [1.34, 1.74]), angina or coronary heart disease (AOR = 1.43, 95% CI [1.25, 1.65]), and stroke (AOR = 1.62, 95% CI [1.42, 1.85]). Those with a behavioral risk score of 1 also had significantly higher odds of having increased odds of reporting a heart attack (AOR = 1.27, 95% CI [1.11, 1.45]), angina or coronary heart disease (AOR = 1.22, 95% CI [1.07, 1.39]), and stroke (AOR = 1.31, 95% CI [1.15, 1.50]).

Compared to individuals aged 18 to 24, the odds of experiencing a heart attack increased significantly with age. Participants aged 35 to 44 had more than twice the odds (AOR = 2.94; 95% CI [1.83, 4.71]), while those aged 45 to 54 had over five times the odds (AOR = 5.98; 95% CI [3.78, 9.44]). The risk continued to rise among participants aged 55 to 64 (AOR = 8.98; 95% CI [5.71, 14.13]) and was the highest in those aged 65 and older (AOR = 11.22; 95% CI [7.17, 17.57]). These findings indicate a strong association between increasing age and heart attack risk beginning at age 35.

The odds of CHD or angina also varied notably by age. Compared to individuals aged 18 to 24, the odds were significantly higher among those aged 45 to 54 (AOR = 3.47; 95% CI [2.30, 5.25]), 55 to 64 years (AOR = 5.21; 95% CI [3.47, 7.82]) and highest among those aged 65 years and older (AOR = 7.38; 95% CI [4.93, 11.06]). A similar age-related trend was observed for stroke. Relative to the 18 to 24 age group, the odds of stroke increased steadily across age brackets: 35 to 44 (AOR = 3.28; 95% CI [2.17, 4.96]), 45 to 54 (AOR = 5.74; 95% CI [3.84, 8.60), 55 to 64 (AOR = 6.74; 95% CI [4.52, 10.06]), and ≥65 years (AOR = 6.97; 95% CI [4.71, 10.3]). Sex differences were also significant, with men having higher odds than women for heart attack (AOR = 2.15; 95% CI [2.00, 2.32]), CHD/angina (AOR = 1.91; 95% CI [1.78, 2.04]), and stroke (AOR = 1.20; 95% CI [1.12, 1.29]).

Using non-Hispanic White adults as the reference group, the odds of heart attack were significantly lower among non-Hispanic Black adults (AOR = 0.76; 95% CI [0.66, 0.87]). Similarly, the odds of CHD or angina were significantly lower among Black, non-Hispanic adults (AOR = 0.82; 95% CI [0.73, 0.94]) and Hispanic adults (AOR = 0.687 95% CI [0.58, 0.77]). However, the risk of stroke varied by race and ethnicity. Non-Hispanic Black adults (AOR = 1.27; 95% CI [1.14, 1.43]) and non-Hispanic multiracial (AOR = 1.38; 95% CI [1.16, 1.64]) had significantly higher odds of stroke, whereas Hispanic adults had lower odds (AOR = 0.77; 95% CI [0.66, 0.89]) compared to non-Hispanic White adults.

Marital status and educational attainment were both associated with differences in CVD risk. Compared to married or cohabitating individuals, previously married participants had higher odds of experiencing a heart attack (AOR = 1.17; 95% CI [1.08, 1.26]), while never-married individuals had lower odds (AOR = 0.85; 95% CI [0.74, 0.98]). The odds of CHD or angina were also lower among never-married individuals (AOR = 0.79; 95% CI [0.68, 0.92]). For stroke, previously married participants had increased odds (AOR = 1.23; 95% CI [1.14, 1.34]).

Education level was another significant factor. Compared to participants who had less than a high school education, the odds of experiencing a heart attack were significantly lower for participants who were high school graduates (AOR = 0.73; 95% CI [0.64, 0.82]), those with some college education (AOR = 0.70; 95% CI [0.62, 0.80]), and college graduates (AOR = 0.53; 95% CI [0.46, 0.61]). Participants with college education had lower odds of experiencing stroke compared to those with less than a high school education (AOR = 0.77; 95% CI [0.67, 0.88]).

Housing and employment status were both associated with CVD outcomes. Compared to homeowners, participants who rented house had higher odds of experiencing a heart attack (AOR = 1.23; 95% CI [1.11, 1.36]) and stroke (AOR= 1.20, 95% CI [1.08, 1.32].; however, no significant differences were observed for CHD based on housing status; CHD or Angina (AOR = 1.09, 95% CI [0.98, 1.20].

Employment status showed stronger associations. Relative to employed adults, the odds of a heart attack were significantly higher for those who were unemployed (AOR = 1.63; 95% CI [1.48, 1.79]). Similar trends were observed for CHD/angina: unemployed (AOR = 1.97; 95% CI [1.79, 2.17]), 3.50]). Stroke risk followed the same pattern, with elevated odds for the unemployed (AOR = 2.21; 95% CI [1.99, 2.46]),

Compared with participants earning below $50,000, those with higher incomes had significantly lower odds of CVD outcomes. Individuals earning $50,000–$99,999 had reduced odds of heart attack (AOR = 0.69; 95% CI [0.61, 0.79]), CHD or angina (AOR = 0.76, 95% CI [0.67, 0.87] and stroke (AOR = 0.66; 95% CI [0.58, 0.75]). The odds were even lower among those earning $100,000 or more for heart attack (AOR = 0.60; 95% CI [0.52, 0.69]), CHD or angina (AOR = 0.70, 95% CI [0.61, 0.81] and stroke (AOR = 0.53; 95% CI [0.46, 0.62]).

Our study showed that high cholesterol was associated with increased risks of heart attack (AOR = 1.78; 95% CI [1.65, 1.92]), CHD/angina (AOR = 2.16; 95% CI [2.01, 2.33]), and stroke (AOR = 1.44; 95% CI [1.33, 1.56]). Similarly, participants with high blood pressure had markedly higher odds of all three outcomes: heart attack (AOR = 2.25; 95% CI [2.08, 2.44]), CHD/angina (AOR = 2.37; 95% CI [2.18, 2.57]), and stroke (AOR = 2.28, 95% CI [2.09, 2.49]).

Compared to participants who do not have some form of health insurance, participants having some form of insurance had higher odds of CHD/Angina (AOR = 1.38, 95% CI [1.09,1.74]) and stroke (AOR = 1.27, 95% CI [1.03,1.56]).

## 4. Discussion

This study used a retrospective cross-sectional study design, using nationally representative data to analyze the association of a composite score of risky health behaviors such as smoking, alcohol use, and physical inactivity, as well as other covariates, with CVD. Our multivariable logistic regression analyses show significant associations between the number of behavioral risks and the odds of reporting each CVD, such as heart attack, angina, coronary heart disease (CHD), and stroke. The findings with adjusted odds ratios over 1.5 (or less than 0.67) in our study reflect stronger connections. Participants reporting one or more behavioral risk factors were more likely to report these CVDs than those without any of those risky health behaviors. The risk of each CVD increased incrementally with the increase in the behavioral risk factor score. These findings highlight the innovation in concurrently addressing various health behaviors for their association with the burden of cardiovascular disease. This combined measure more comprehensively depicts the cumulative burden of lifestyle risk factors and reflects the frequent clustering of such behaviors within individuals. We have observed a distinct “dose–response” pattern in our results, which indicates that the likelihood of cardiovascular disease increases gradually as the number of co-occurring risk behaviors increases. This integrated approach enhances previous research by offering a more comprehensive measure of behavioral risk clustering and its compounded relationship with cardiovascular outcomes in the adult population of the United States.

Although other studies did not examine the interaction of multiple behavior risk factors, our composite score findings are consistent with previous studies—for instance, Barbaresko et al., who conducted the meta-analysis of 22 studies, found that 66% of the risk of CVDs can be decreased with a healthy behaviors, such as regular physical activity, no smoking, and no alcohol consumption [41]. Tsai et al. also analyzed the young and older adult population in the US, showing that healthy behaviors like no smoking, moderate alcohol consumption, regular physical activity, etc., have a lower risk of CVDs with a pooled hazard ratio of 0.37 (95% CI 0.31–0.43) [42]. A systematic review and meta-analysis of prospective cohort studies included many studies to see the relationship of combined lifestyle factors and all-cause and CVD mortality. Like our study, risk factors, such as lack of physical activity, smoking, heavy alcohol consumption, and other factors, were studied. Findings suggested that people with the healthiest lifestyle had lower mortality risk for CVDs and all-cause mortality [43].

Heart disease has remained a leading cause of mortality and morbidity in the U.S. since the 1950s [44], and our study confirms that the risk of CVD increased with age. This finding aligns with previous research from over a decade ago, which also identified advancing age as a major risk factor for CVD [45,46]. These trends carry significant public health implications, especially given the projected 9.4% growth in the U.S. population aged 65 and older between 2020 and 2023 [47]. By 2030, older adults are expected to comprise 20% of the total U.S. population, approximately 71 million people [48].

Our findings are consistent with previous studies that analyzed BRFSS data, which have consistently shown CVD-related mortality increases with age in the U.S. population [49,50,51]. Consistent with previous research, we also found that men are at a higher risk of developing CVDs as compared to women [52,53]. One possible explanation is the protective effect of endogenous estrogen during the reproductive years, which may reduce the relative risk of hypercholesterolemia and contribute to the lower prevalence of CVDs among women [54].

Our study also indicated lower odds of CVD among Black participants. However, this contrasts with research by Kyalwazi AN et al., which reported higher CVD-related mortality among Black adults compared to White adults [55]. One possible explanation is provided by a separate study, which found that while the prevalence of risk factors is higher in the Black population, the rates of major adverse cardiovascular events, such as epicardial coronary artery disease, were actually lower compared to their White counterparts [56]. National statistics from 2019 also showed a lower prevalence of coronary heart disease or angina among Black adults (10%) compared to White adults (13.9%), as well as a lower prevalence of heart attack (10.7% vs. 14.7%) [39].

Additionally, our study found that previously married people are at higher risk of developing CVDs. However, this contrasts with findings from a meta-analysis by Wong et al., which reviewed 34 studies involving over two million participants and concluded that being unmarried, regardless of marital history, was associated with higher odds of developing CVD [57].

Our study found that high school graduates and individuals with annual incomes greater than $25,000 have a lower risk of developing CVDs. This aligns with findings from the 2006–2014 National Death Index-linked National Health Interview Survey, which reported that adults with less than a high school education faced a 20% to 40% increased risk of mortality from heart-related conditions [58]. Similarly, Minhas et al. demonstrated that lower family income was associated with higher odds of all-cause mortality and cardiac mortality [59].

Our study also found that housing status is associated with CVDs, with individuals living in rented homes having a higher risk of developing the disease compared to homeowners. This finding aligns with previous research identifying homeownership as a protective factor for the risk of angina and CHD, likely due to the financial stability it provides. In contrast, financial hardship is more common among renters [60,61].

Employment status also plays a significant role. Consistent with earlier studies linking unemployment and job loss to acute heart conditions [62], our results show that unemployed individuals are at greater risk for CVDs. Similarly, Elfassy et al. found that greater income volatility and substantial income loss were associated with a higher risk of CVDs and all-cause mortality [63].

Interestingly, our analysis also showed lower CVD odds among overweight participants. However, this finding diverges from existing literature, which has consistently shown that being overweight is associated with increased risk for heart disease [64,65]. Further research is needed to better understand the complex relationship between body weight and cardiovascular outcomes.

Furthermore, our study highlights a strong association between high blood pressure and increased odds of CVD, in line with more recent studies that confirm this relationship [66,67]. Elevated cholesterol was also significantly associated with CVDs in our analysis. This supports findings from the Cooper Center Longitudinal Study, which showed that high levels of LDL-C and non–HDL–C (≥160 mg/dL) are associated with increased CVD mortality [68].

Our finding that people with health insurance had an association with CHD/angina and stroke is consistent with previously conducted studies. That study concluded that treatment and control of heart disease risk factors were lower in the uninsured adult population [69]. Our study found the interaction of risk behaviors in the development of CVDs. When we combined the physical activity, smoking status, and consumption of heavy alcohol in the study participants, our study found that the progression of CVDs occurs as the number of risk factors increases. If there is smoking with heavy alcohol consumption or lack of exercise, study participants are at potential risk of developing CVDs. Similarly, if there are two or more risk behaviors present in the participants, the risk of developing CVDs further increases.

One of the apparently counterintuitive findings of our study was the absence of a significant association between reported obesity/overweight and CVD. While this may be caused by misclassification in self-reported height and weight (discussed later among limitations), the obesity paradox may also explain it. It occurs in chronic conditions, where persons who are overweight or mildly obese (BMI 25–30 kg/m^2^) have better survival results than those with normal or low body weight. This contradiction occurs in heart failure, chronic renal illness, and type 2 diabetes. Recent research suggests that mildly raised BMI may improve mortality in select populations (e.g., those with advanced diabetic nephropathy), although both underweight and obesity statuses increase the risk of CVD [70]. These findings demonstrate the limitations of BMI alone and the need for a more complete body composition assessment. In addition, although the absence of a statistically significant connection between obesity and CVD may seem contradictory, it highlights the intricate interaction of behavioral and metabolic factors. A longitudinal study is better-suited to examine whether diseases, including hypertension, hypercholesterolemia, and diabetes, mediate lifestyle choices and cardiovascular outcomes. These studies would clarify causal directions and interrelationships.

It is important to acknowledge several limitations of this study, which may affect the generalizability of our findings. First, like other cross-sectional surveys conducted in the US, BRFSS is undertaken at one point in time. Therefore, our cross-sectional study results should imply associations but cannot determine the cause-and-effect relationship between the CVDs and risk behaviors. Our study also shows only the association of risk behaviors with CVDs. Still, it cannot establish that the associated risk behaviors are the causes of the CVDs in the US adult population. Second, both dependent and independent variables in our study were based on self-reported behaviors and the presence of CVD, rather than clinical assessments. Some participants may not be aware of their chronic conditions because their conditions were not diagnosed. A potential for misclassification of health conditions, such as high cholesterol or COPD, also existed, as participants may inaccurately understand or report their diagnosis. Height and weight are also self-reported by the participants in the BRFSS survey, so inaccurate height and weight may cause miscalculation of the BMI in the US adult population, resulting in the true association between Obesity and CVDs. Perhaps this explains why obesity and cardiovascular disease are not statistically associated after statistically controlling for health risk factors for both obesity and cardiovascular disease. Third, the risk of recall bias is frequently found in self-reported data. There is a potential risk of recall bias in the BRFSS 2023 data, specifically for the self-reported data of High Blood Pressure and High Cholesterol variables. This can either distort or weaken the associations between variables, with the potential for recall bias and CVDs. Fourth, the social desirability may also alter responses about undesirable behaviors such as smoking, alcohol use, and a sedentary lifestyle. Social desirability bias may have led participants to overreport positive behaviors and underreport undesirable conditions. BRFSS collects data about health behaviors like physical activity, exercise, smoking, and heavy alcohol consumption. Due to social desirability bias, participants may overreport high physical activity than they perform. On the other hand, participants may underreport or not report their heavy alcohol consumption or their smoking status. Participants may also underreport their chronic heart conditions due to social desirability bias in society.

Despite these limitations, our study has several noteworthy strengths that enhance the value of the findings. It draws on data from a large, nationally representative sample covering all U.S. states and territories, allowing for a robust analysis across diverse demographic and socioeconomic groups. The survey’s well-tested design provides a comprehensive view of public health, capturing trends in health behaviors, chronic conditions, mental health, opioid use, and social determinants of health. Furthermore, the data’s applicability at the local, state, and regional levels enhances its relevance for public health policy and targeted intervention planning.

The use of complex sampling weights ensures that underrepresented groups are adequately included, improving the generalizability of the findings to a broader adult U.S. population. Taken together, these strengths underscore the value of BRFSS data for advancing understanding of cardiovascular disorders and their risk factors, while reinforcing the importance of our findings for informing prevention strategies and public health policy.

## 5. Conclusions

This study examines how unhealthy behaviors and social determinants are associated with the risk of CVD among adults in the U.S., offering practical evidence to guide public health practice and policy. The findings indicate that several sociodemographic factors, such as age, male gender, marital disruption, unemployment, and homeownership, are significantly associated with increased risk of heart attack, CHD, and stroke. Additionally, the combined score of behavioral risk factors such as smoking, alcohol use, and physical inactivity, along with clinical conditions like hypertension and hyperlipidemia, further increases the odds of CVD. These results highlight substantial health disparities, suggesting that social and economic inequities, particularly those related to geographic location and income, play a critical role. For example, U.S. adults living in nonmetropolitan counties or belonging to lower-income groups face a disproportionately higher risk of CVD. The implications for public health are considerable. Interventions and policies must aim not only to improve overall population health but also to specifically target modifiable risk factors like smoking, blood pressure, cholesterol, and physical inactivity, particularly among underserved and marginalized groups. Notably, the findings emphasize the importance of addressing upstream social and economic inequities to improve downstream health outcomes. Effective public health strategies should move beyond individual behavior and instead leverage social systems, infrastructure, and community resources to deliver holistic and culturally sensitive interventions. Public health practice initiatives should integrate behavior-centered health promotion approaches with policies that directly address socioeconomic and geographic disparities to ensure equitable improvements in CVD.

## Figures and Tables

**Table 1:** Frequency (unweighted), Percentage (Weighted), and Lower and Upper Confidence Intervals.

Response	N (Unweighted)	Percent	Lower CL	Upper CL
		(Weighted)		
Heart Attack (n = 430,755)				
Yes	23,451	4.19	4.07	4.32
No	407,304	95.81	95.68	95.93
Total	430,755	100	.	.
Don’t know/Not sure	2314			
Refused	251			
Missing	3			
Angina or Coronary Heart Disease (n = 429,092)				
Yes	23,454	4.07	3.95	4.18
No	405,638	95.93	95.82	96.05
Total	429,092	100	.	.
Don’t know/Not sure	3936			
Refused	292			
Missing	3			
Stroke (n = 431,849)				
Yes	18,350	3.39	3.28	3.5
No	413,499	96.61	96.5	96.72
Total	431,849	100	.	.
Don’t know/Not sure	1212			
Refused	258			
Missing	4			
**Controlled Variables (Age, Gender, Race, Marital Status, Education, Employment, Income, Ownership of home, and health insurance)**				
Imputed Age in Six Groups (n = 433,323)				
Age 18 to 24	26,281	12.37	12.12	12.63
Age 25 to 34	46,077	16.5	16.24	16.76
Age 35 to 44	56,224	16.44	16.18	16.69
Age 45 to 54	61,848	15.21	14.97	15.46
Age 55 to 64	77,938	15.93	15.69	16.18
Age 65 or older	164,955	23.54	23.3	23.79
Total	433,323	100	.	.
Gender (n = 433,323)				
Female	229,523	51.5	51.16	51.84
Male	203,800	48.5	48.16	48.84
Total	433,323	100	.	.
Five level race/ethnicity category (n = 433,237)				
White Only, Non-Hispanic	313,067	55.63	55.3	55.96
Black Only, Non-Hispanic	32,919	11.69	11.46	11.91
Other Race Only, Non-Hispanic	24,356	8.36	8.12	8.6
Multiracial, Non-Hispanic	10,125	2.68	2.57	2.79
Hispanic	43,286	19.34	19.03	19.64
Don’t Know/Not Sure/Refused	9484	2.31	2.21	2.41
Total	433,237	100	.	.
Missing	86			
Marital Status (n = 433,316)				
Married or Cohabitating	241,362	54.8	54.47	55.13
Previously Married	110,548	19.41	19.17	19.66
Never Married	77,124	24.66	24.36	24.96
Refused/Don’t Know/Not Sure	4282	1.13	1.05	1.2
Total	433,316	100	.	.
Missing	7			
Education Level (n = 433,314)				
Less than High School	25,172	11.57	11.31	11.83
High School Graduate	106,613	27.04	26.75	27.34
Some College	114,346	29.9	29.58	30.21
College Graduate or Higher	184,867	30.9	30.61	31.18
Refused	2316	0.59	0.54	0.65
Total	433,314	100		
Missing	9			
Employment Status (n = 430,355)				
Employed	215,794	56.22	55.89	56.55
Unemployed	16,644	5.4	5.23	5.56
Not in the Workforce	167,714	30.73	30.43	31.02
Unable to Work	25,490	6.25	6.09	6.41
Refused	4713	1.41	1.32	1.5
Total	430,355	100	.	.
Missing	2968			
Computed Employment Status (n = 430,355)				
Employed	215,794	56.22	55.89	56.55
Unemployed	214,561	43.78	43.45	44.11
Total	430,355	100	.	.
Missing	2968			
Income Level (n = 433,323)				
Less than $25,000	50,256	12.42	12.19	12.64
$25,000 to $49,999	86,010	19.3	19.04	19.56
$50,000 to $99,999	107,027	22.29	22.01	22.56
$100,000 or More	103,407	24.27	23.99	24.56
Refused/Don’t Know/Not Sure/Missing	86,623	21.73	21.44	22.01
Total	433,323	100	.	.
Own or Rent Home (n = 433,315)				
Own	301,956	66.89	66.59	67.18
Rent	105,960	25.59	25.32	25.85
Refused/Don’t Know/Not Sure	25,399	7.53	7.34	7.71
Total	433,315	100	.	.
Missing	8			
Health Insurance (n = 433,323)				
Do Not Have Some Form of Health Insurance	22,703	8.11	7.9	8.33
Have Some Form of Insurance	391,946	86.31	86.05	86.57
Don’t Know, Refused, or Missing Insurance Response	18,674	5.58	5.41	5.74
Total	433,323	100	.	.
**Modifiable Risk Behaviors (Exercise, Smoking, and Heavy Drinking**				
Physical Activity (n = 433,323)				
Inactive	108,506	25.14	24.85	25.44
Highly Active	156,975	33.01	32.7	33.33
Active	49,274	11.95	11.73	12.16
Insufficiently Active	33,587	9.22	9.03	9.42
Don’t know/Not Sure/Refused/Missing	84,981	20.67	20.4	20.95
Total	433,323	100	.	.
Computed Smoking Status (n = 433,323)				
Never Smoked	251,981	60.93	60.6	61.25
Current Smoker—Now Smokes Every Day	31,770	7.24	7.08	7.41
Current Smoker—Now Smokes Some Days	13,376	3.44	3.31	3.56
Former Smoker	113,134	22.15	21.89	22.42
5: Don’t Know/Refused/Missing	23,062	6.25	6.08	6.42
Total	433,323	100	.	.
Heavy Alcohol Consumption Calculated Variable (n = 433,323)				
No	376,780	85.67	85.43	85.91
Yes	23,718	5.19	5.05	5.33
Don’t Know/Refused/Missing	32,825	9.14	8.93	9.34
Total	433,323	100	.	.
Behavioral Score				
0 (no smoking/no heavy drinking/exercise present)	276,710	64.45	64.12	64.77
1 (only one risk factor)	127,590	29.95	29.63	30.26
2 (two or more risk factors)	24,028	5.61	5.45	5.76
Total	428,328	100	.	.
**Morbidities (Overweight or obese, High Blood Pressure, High Cholesterol)**				
Overweight or Obese Calculated Variable (n = 433,323)				
No	123,267	28.92	28.61	29.23
Yes	269,521	60.15	59.82	60.48
Don’t Know/Not Sure/Refused	40,535	10.93	10.71	11.16
Total	433,323	100	.	.
High Blood Pressure Calculated Variable (n = 433,323)				
No	255,182	65.2	64.89	65.51
Yes	176,222	34.28	33.97	34.59
Don’t Know/Not Sure/Refused/Missing	1919	0.52	0.46	0.57
Total	433,323	100	.	.
High Cholesterol Calculated Variable (n = 433,323)				
No	219,333	52.22	51.89	52.56
Yes	158,906	31.36	31.05	31.66
Don’t Know/Not Sure/Refused/Missing	55,084	16.42	16.16	16.68
Total	433,323	100	.	.

Note: Variations in n are due to item non-response. Abbreviations: CL—confidence interval; n—number of observations.

**Table 2:** Multivariable Logistic Regression of Self-Reported Heart Attack, Angina or CHD, and Stroke (vs. not Reported).

	Heart Attack			Angina or CHD			Stroke		
		CL			CL			CL	
Variables	AOR	Lower	Upper	AOR	Lower	Upper	AOR	Lower	Upper
Behavior Score									
Behavior score 1	**1.35**	1.25	1.45	**1.24**	1.16	1.34	**1.37**	1.26	1.48
Behavior score 2	**1.53**	1.34	1.74	**1.43**	1.25	1.65	**1.62**	1.42	1.85
Reference Behavior Score = 0 *									
Age Group									
Age 25 to 34	**1.91**	1.14	3.19	1.39	0.85	2.26	**1.64**	**1.05**	**2.57**
Age 35 to 44	**2.94**	1.83	4.71	**1.82**	1.18	2.81	**3.28**	2.17	4.96
Age 45 to 54	**5.98**	3.78	9.44	**3.47**	2.30	5.25	**5.74**	3.84	8.60
Age 55 to 64	**8.98**	5.71	14.13	**5.21**	3.47	7.82	**6.74**	4.52	10.06
Age 65 or older	**11.22**	7.17	17.57	**7.38**	4.93	11.06	**6.97**	4.71	10.30
Age 18 to 24 (Reference group) *									
Gender									
Male	**2.15**	2.00	2.32	**1.91**	1.78	2.04	**1.20**	1.12	1.29
Female (Reference group) *									
Race/Ethnicity									
Black only, Non-Hispanic	**0.76**	0.66	0.87	**0.82**	0.73	0.94	**1.27**	1.14	1.43
Other race only, Non-Hispanic	0.85	0.73	1.00	0.99	0.82	1.19	1.02	0.85	1.22
Multiracial, Non-Hispanic	**1.27**	1.04	1.54	1.10	0.90	1.33	**1.38**	1.16	1.64
Hispanic	**0.81**	0.70	0.93	**0.67**	0.58	0.77	**0.77**	0.66	0.89
Don’t know/Not sure/Refused	1.02	0.77	1.35	1.13	0.86	1.49	1.19	0.88	1.59
White only, Non-Hispanic *									
Marital Status									
Previously Married	**1.17**	1.08	1.26	1.03	0.95	1.12	**1.23**	1.14	1.34
Never married	**0.85**	0.74	0.98	**0.79**	0.68	0.92	0.98	0.84	1.13
Refused/Don’t know/Not sure	0.83	0.56	1.22	0.79	0.54	1.14	**0.57**	0.42	0.79
Married or Cohabitating *									
Education Level									
High School Graduate	**0.73**	0.64	0.82	**0.87**	0.76	0.99	0.91	0.81	1.04
Some College	**0.70**	0.62	0.80	**0.86**	0.76	0.99	0.98	0.86	1.11
College Graduate or Higher	**0.53**	0.46	0.61	**0.75**	0.65	0.86	**0.77**	0.67	0.88
Refused/Don’t know/Not sure	0.82	0.54	1.27	1.12	0.58	2.19	0.88	0.35	2.25
Less than High School *									
House Rent/Own Status									
Rent	**1.23**	1.11	1.36	1.09	0.98	1.20	**1.20**	1.08	1.32
Refused/Don’t know/Not sure	1.07	0.93	1.25	1.10	0.93	1.31	**1.20**	1.03	1.39
Own *									
Employment Status									
Unemployed	**1.63**	1.48	1.79	**1.97**	1.79	2.17	**2.21**	1.99	2.46
Employed *									
Income Level									
$25,000 to $49,999	**0.89**	0.81	0.99	**0.85**	0.76	0.94	**0.88**	0.79	0.98
$50,000 to $99,999	**0.69**	0.61	0.79	**0.76**	0.67	0.87	**0.66**	0.58	0.75
$100,000 or more	**0.60**	0.52	0.69	**0.70**	0.61	0.81	**0.53**	0.46	0.62
Refused/Don’t know/Not sure	**0.75**	0.67	0.84	**0.74**	0.66	0.83	**0.75**	0.66	0.84
Less than $25,000 *									
Overweight or Obese Calculated									
Yes	0.99	0.91	1.07	1.03	0.95	1.12	0.93	0.86	1.01
Don’t know/Not sure/Refused	0.86	0.72	1.03	0.93	0.80	1.07	0.81	0.70	0.95
No									
High Cholesterol (Calculated)									
Yes	**1.78**	1.65	1.92	**2.16**	2.01	2.33	**1.44**	1.33	1.56
Don’t know/Not sure/Refused/	1.10	0.95	1.27	0.99	0.85	1.16	**1.19**	1.03	1.37
No									
High Blood Pressure (Calculated)									
Yes	**2.25**	2.08	2.44	**2.37**	2.18	2.57	**2.28**	2.09	2.49
Don’t know/Not Sure/Refused/Missing	**2.11**	1.11	4.01	1.49	0.84	2.66	1.45	0.82	2.57
No									
Health Insurance Status									
Have some form of insurance	1.08	0.89	1.30	**1.38**	1.09	1.74	**1.27**	1.03	1.56
Don’t know, refused or missing insurance response	1.23	0.94	1.62	1.29	0.95	1.74	**1.38**	1.07	1.79
Do not have some form of health insurance *									

Abbreviations: AOR—adjusted odds ratio; CL—confidence interval; CHD—coronary heart disease. Note: The following are number of observations (n) per model, after exclusions of missing observations: n = 400,096 for heart attack; n = 421,923 for CHD; n = 424,626 for stroke. Pseudo-R^2^ (McFadden’s) = 0.175 for heart attack, pseudo-R^2^ = 0.185 for CHD, and pseudo-R^2^ = 0.144 for stroke. * indicates the reference category. The AORs in bold indicate the significance of differences relative to the reference category at *p* ≤ 0.05.

## Data Availability

This study used data from 2023 BRFSS Survey. Data and Documentation can be downloaded at: https://www.cdc.gov/brfss/annual_data/annual_2023.html, accessed on 18 January 2025. No new or any other data collected and used in this study. The authors did not have special access that others would not have.

## Abbreviations

The following abbreviations are used in this manuscript:

AOR	Adjusted odds ratio
AUD	Alcohol use disorder
BRFSS	Behavioral risk factor surveillance system
CDC	Centers for disease control and prevention
CHD	Coronary heart disease
CI	Confidence interval
COPD	Chronic obstructive pulmonary disease
CVDs	Cardiovascular diseases
DALY	Disability-adjusted life years
NCDs	Non-communicable diseases
NHANES	National health and nutrition examination survey
US	United states
WHO	World health organization
